# Obstructive Form of Hypertrophic Cardiomyopathy-Left Ventricular Outflow Tract Gradient: Novel Methods of Provocation, Monitoring of Biomarkers, and Recent Advances in the Treatment

**DOI:** 10.1155/2016/1575130

**Published:** 2016-05-10

**Authors:** Pawel Petkow Dimitrow, Renata Rajtar-Salwa

**Affiliations:** 2nd Department of Cardiology, Jagiellonian University Collegium Medicum, Kopernika Street 17, 31-501 Cracow, Poland

## Abstract

Dynamic (latent or/and labile) obstruction of left ventricular outflow (LVOT) was recognized from the earliest clinical descriptions of hypertrophic cardiomyopathy (HCM) and has proved to be a complex phenomenon, as well as arguably the most audible (“visible”) pathophysiological hallmark of this heterogeneous disease. The aim of the current review is focused on two novel issues in a subgroup of obstructive HCM. Firstly, the important methodological problem in HCM is the examination of a subgroup of patients with nonobstructive hypertrophy in resting conditions and hard, but possible provoking obstruction. Recently, investigators have proposed physiological stress test (with double combined stimuli) to disclose such type of patients. The upright exercise is described in the ESC guideline on hypertrophic cardiomyopathy from 2014 and may appear as a candidate for gold standard provocation test. The second novel area of interest is associated with elevated level of signaling biomarkers: hypercoagulation, hemolysis, acquired von Willebrand 2A disease, and enhanced oxidative stress. The accelerated and turbulent flow within narrow LVOT may be responsible for these biochemical disturbances. The most recent advances in the treatment of obstructive HCM are related to nonpharmacological methods of LVOT gradient reduction. This report extensively discusses novel methods.

## 1. Introduction

History of recognition of hypertrophic cardiomyopathy (HCM) and definition of frequency of obstructive form is associated also with methodology of stress test to provoke LVOT gradient >30 mmHg. Maximized and (semi)physiological stress tests were introduced only several years ago.

**Figure 1:**
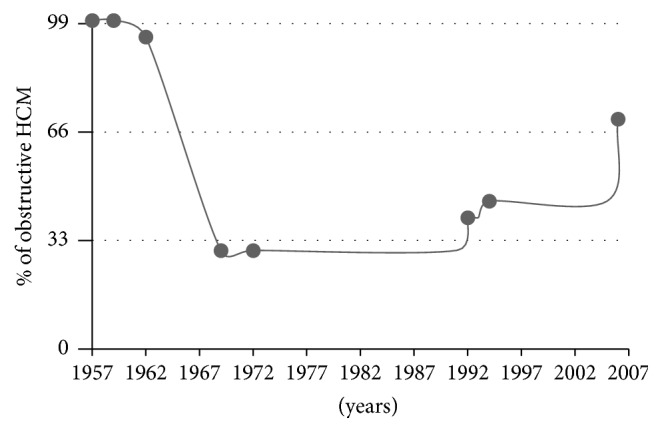
Probable timeline displaying major events concerning the history of left ventricular outflow tract obstruction in hypertrophic cardiomyopathy. Point 1: first description of obstructive HCM by Brock. Point 2: Morrow and Braunwald (1959) and Braunwald et al. (1960): all patients were defined as obstructive HCM by systolic murmur in auscultation and LVOT gradient in invasive catheterization. Point 3: Braunwald et al.  (1962, 1963), description of first HCM patient without obstruction. Point 4: first echocardiographic image of SAM by Shah et al. Point 5: echocardiographic description of asymmetrical left ventricular hypertrophy by Henry et al. Point 6: description of using of DDD pacing. Point 7: description of intervention defined as alcohol septal ablation. Point 8: publication by Maron et al. describing exercise test for LVOT gradient provocation.

For more than 50 years, in the period of first description among alive patients (in preechocardiographic era, Figure 1), HCM was only exclusive as obstruction, where systolic murmur in auscultation was verified as turbulent flow with gradient in obstructive LVOT measured invasively [1–3]. In fact, in the early preechocardiographic era (1957 to 1969), an outflow gradient was a virtual prerequisite for the diagnosis of HCM. Dynamic obstruction to LVOT rapidly achieved distinction as the most represented feature of HCM, dominating the initial comprehensive description of the disease.

Thus, predominantly, but not solely [4, 5], obstructive HCM has been diagnosed in early reports. After introduction of echocardiography [6–8] (Figure 1), during family screening for sudden cardiac death, a large number of nonobstructive patients were recognized. In this period researchers postulated that, in the whole population of HCM, the ratio of the obstructive: nonobstructive is 1/3 : 2/3. In 2003 it was reported that, among older patients, female's left ventricular contractility was higher and LVOT gradient occurred more frequently than in males [9]. Recent observation that the vast majority of patients with HCM have the propensity to develop outflow obstruction (either at rest or with exercise) underscores a return to the characterization of HCM in early period of exploration as a predominantly obstructive disease [10].

To sum up the history, our perception of obstructive : nonobstructive ratio has changed during the last 55 years. In Figure 1 we can recognize early period of obstructive predominance, midperiod with nonobstructive advantage due to broad availability of echocardiography, and later period with increasing frequency of obstructive form up to value 70% [10]. The low percent of obstruction proportions started to reverse during the nineties. With the introduction of less invasive than myotomy-myectomy, but nonpharmacological therapies, DDD pacing [11] and alcohol septal ablation [12] were associated with more selected groups of patients (referral bias) and increasing types and numbers of provocative maneuvers. More varied stimuli were needed to more adequately qualify patients with obstruction.

## 2. History of LVOT Provoking Stimuli

**Figure 2:**
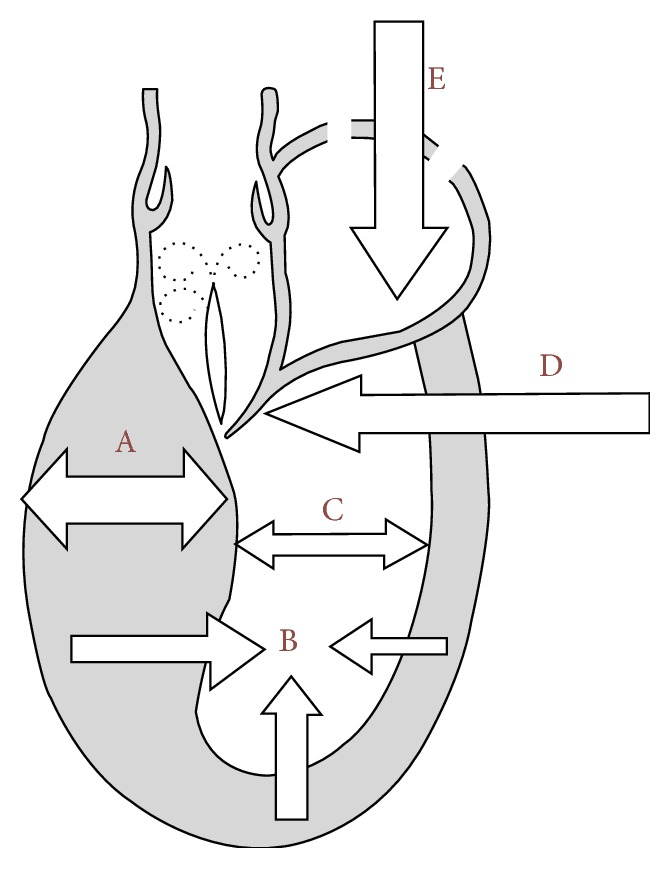
Mechanisms predisposing to LVOTG induction. A: left ventricular hypertrophy, particularly basal septal segment (HCM, hypertension, and storage disease). B: LV hypercontractility (moderate tachycardia). C: small size LV cavity (HCM, children, women, dehydration). D: prolonged/thickened mitral leaflet(s). E: reduced LV preload (dehydration, diuretics, vasodilators, hemodialysis, fever, and septic shock).

Obstruction in HCM has been featured by dynamic nature [13, 14], in which pressure gradients can vary considerably with a variety (nonstandardized methodology) of pharmacological and physiological provocations that reduce peripheral arterial pressure or ventricular volume or enhance myocardial contractility and may change even after heavy meals or alcohol intake or spontaneously on a day-to-day or hour-to-hour basis [10, 13–21]. Mechanisms predisposing to LVOT gradient induction are presented in Figure 2. It was recognized that dynamic outflow gradients could be provoked by physiological exercise or a variety of nonphysiological maneuvers, including sympathomimetic agents, such as infused isoproterenol or dobutamine, or by introducing premature ventricular beats, amyl nitrite inhalation or nitroglycerin, and the Valsalva maneuver [10, 13–21]. The ground breaking point occurred in 2006, when Maron et al. proposed exercise as a provocation and therefore the obstructive group was as numerous as 70% [10].

Importantly, this study included a large number of patients. However, there are some disadvantages of the proposed method. The gradient provoked by exercised stress test was measured with delay in postexercise recovery and in semisupine position, instead of at peak exercise in upright position.

**Figure 3:**
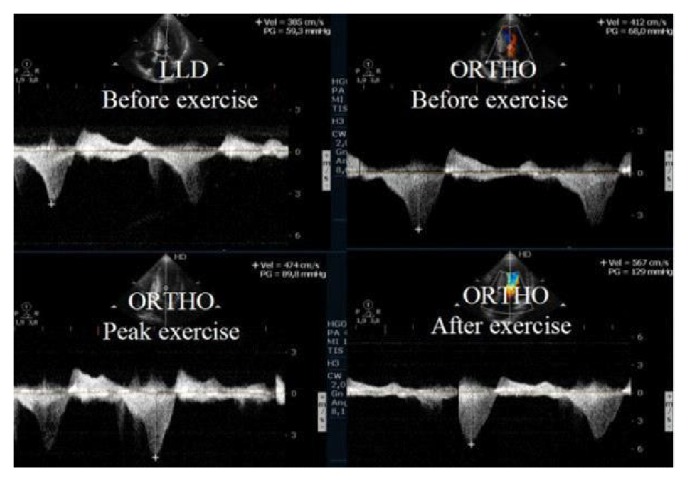
Intraventricular gradient present in all phases of the study in a HCM patient with increase also after exercise in orthostatic position.

Earlier, in small selected group of patients, German [22] and Portuguese [23] investigators proposed pioneering full-upright exercise test. Researchers began to consider that we need natural, strong but physiological stimuli. The exercise test to provoke gradient has been postulated as a gold standard. Thus, the latest step of development was to combine two (perfectly physiological) stimuli, that is, upright position (reduced LV preload) and exercise by ergometer or treadmill [18, 20–31] (Figure 3). Standing position, both at rest and during exercise, is a normal and fundamental activity of daily life but may precipitate an unexpected fall in cardiac patients predisposed to syncope, especially in patients with unsuspected LVOT obstruction. Recently, a novel interesting combination was use of exercise test with postprandial status which additionally reduces LV preload [32].

In the most recent report, other types of stimuli have been combined—nitroglycerine and Valsalva to maximize provocation [33]. Proposed procedure is less time-consuming and does not require special equipment [33]. Looking for new clinical applications, it has been proposed to use exercise stress echocardiography in the upright position with LVOT gradient monitoring both during and after exercise, as a marker of genotype-positive/phenotype negative hypertrophic cardiomyopathy (prehypertrophic status of HCM) [34]. Upright exercise to provoke (sub)valvular gradient has been used in many cardiac diseases [18].

## 3. Turbulent Flow and Activated Various Biomarkers

Apart from hemodynamic disturbances, obstructive HCM may induce several hematological abnormalities. Abnormalities in blood parameters associated with hemolysis and hypercoagulability status have been recently recognized.

Accordingly, in two Japanese articles [35, 36], the interesting biochemical phenomenon of hemolysis in hypertrophic cardiomyopathy (HCM) patients has been clearly documented, concerning only patients with the obstructive form. In the first case report [35], mechanical intracardiac hemolysis due to LVOT obstruction was advanced and hemolytic anemia recurred. Implanted DDD pacing decreased the LVOT gradient and reduced hemolysis. In the next article, stabile and provoked obstruction was associated with hemolysis [36]. Importantly, for everyday practice, this hemolysis appears to be associated also with LVOT obstruction, only provoked by daily physical activities. Although there was no relation between erythrocyte creatine levels and LVOT gradient in the subgroup of patients without obstruction at rest (before provoked stress test), a correlation between erythrocyte creatine and LVOT gradient provoked by Valsalva became significant in the subgroup of nonobstructive patients. Summing up, patients with higher erythrocyte creatine levels exhibited greater LVOT gradient with Valsalva provocation.

These results suggest that intravascular hemolysis is correlated with severity of LVOT obstruction not at rest, but with daily activities. Importantly, as a clinical implication, this biomarker may be useful for the identification of a subgroup of HCM patients with latent (none easily identified) obstruction. In perspective, we may postulate that measurement of this biomarker appears to be valuable for monitoring and management of HCM with not only stable, but especially interesting labile obstruction.

Hematological abnormalities were described in other studies, which clearly documented increased levels of biomarkers heralding hypercoagulation [37]. Moreover, acquired type of von Willebrand disease (type 2A) [38] has been recognized. It was postulated that rapidly turbulent blood flow within LVOT (narrow, irregular canal) obstruction plays a stimulating role in the activation of various biomarkers and hematological processes.

LVOT obstruction in HCM may provoke procoagulant mechanisms due to high shear stress. Both anatomical (systolic anterior motion of mitral leaflet with or without septal contact) and hemodynamical (high pressure gradient) abnormalities generating turbulent flow might play a role in the prothrombotic state [37]. The precise role of long and thickened mitral leaflets disturbing flow to nonlaminar pattern is an important question for further exploration of hematological disturbances.

Next, pathology in obstructive HCM [38] is the evidence of von Willebrand factor (vWF) impairment as frequent deficiency and closely related to the magnitude of LVOT obstruction. The problem is large in scale because resting LVOT gradient as low as 15 mmHg (common definition of obstruction is >30 mmHg) is absolutely sufficient to impair vWF. The proposed [38] interpretation of hemodynamic-hematologic phenomenon is attractive. Given the unique shear stress characteristics of vWF, it has been postulated that accelerated LVOT velocity at rest or during exercise might increase proteolysis of vWF multimers and impair primary hemostasis in the obstructive HCM. The biological effect of LVOT obstruction on vWF function is detectable in patients with either baseline or latent (exercise-provoked) obstruction. Primary hemostasis assays that assess vWF function impairment strongly correlate with the maximal LVOT gradient and improve with therapeutic intervention reducing gradient. Hemostasis impairment might be responsible for spontaneous bleeding in patients with an obstructive form of HCM.

In conclusion, the authors [38] have demonstrated that high shear forces could induce structural changes in the shape of the vWF molecule, facilitating the action of the specific vWF protease ADAMTS 13, which would lead to the loss of the high-molecular-weight multimer of vWF. Importantly, this process may be reversed by alcohol septal ablation [39] and septal myectomy [40] reducing LVOT gradient. Thus, we are able to correct hematological abnormalities by reduction of LVOT obstruction. In gastrointestinal bleeding as complication of Heyde's syndrome, thalidomide and octreotide therapy can be used to bridge to surgical or alcohol septal ablation [41].

According to other biochemical abnormalities, enhanced oxidative stress [42] and endothelial dysfunction [43] are also characteristics features for obstructive HCM.

## 4. Role of High Troponin

As regards biomarker of ischemic injury of myocardium, levels of serum cTnI are elevated in a significant proportion of obstructive HCM patients [44]. Serum cTnI is associated with multiple parameters of disease severity, suggesting its great significance in assessing cardiac remodeling in patients with obstructive HCM. In obstructive HCM left ventricular hypertrophy is the major determinant of serum cTnI levels [44] and high level of serum cTnT is associated with the presence of AF [45].

Serum cTnI is an independent predictor useful for identifying myocardial fibrosis visualized by late hyperenhancement of gadolinium in cardiac magnetic resonance [46].

## 5. Pharmacological Treatment: Negative Inotropic Effect

According to eminent expert Sherrid [47], inotropic negative effectiveness (power) is categorized as follows: beta-blocker > disopyramide > verapamil. Disopyramide possesses additional beneficial value, that is, vagolytic effect, acting as antibradycardia agent. This positive chronotropic effect of disopyramide may beneficially counterbalance the negative chronotropic effect of beta-blocker and together provide opportunity to safety combination of both drugs for aggressive, but only pharmacological reduction of LVOT gradient.

## 6. Nonpharmacological Option for Treatment

Generally, two invasive methods are strong competitors in current armamentarium. The comparison between surgical myotomy-myectomy and alcohol septal ablation (ASA) is most interesting, whereas DDD pacing is marginalized for rare indication. However in Spanish center [48] beneficial effect has been observed in 18-year-long follow-up study on DDD pacing. Sequential pacing in selected patients with obstructive HCM improves functional class and reduces dynamic gradient and mitral regurgitation immediately after pacemaker implantation and at final follow-up [48]. Prolonged ventricular pacing has no negative effects on systolic or diastolic function in these patients. Both surgical and transcoronary methods have dynamically developed. The long-term survival and clinical outcome of the ASA and surgical method are extensively debated in both USA and Europe. The crucial problem is safety of both invasive methods. Very recently, an important document has appeared [49], analyzing systematic review and meta-analysis of long-term outcomes after invasive septal reduction intervention therapy in obstructive HCM. In large-scale analysis, the authors have compared sixteen cohorts, including 2791 patients after myectomy (mean follow-up: 7.4 years) versus 11 ASA cohorts with 2013 patients (mean follow-up: 6.2 years). Importantly, long-term mortality and (aborted) SCD rates after ASA and myectomy are similar and importantly low. Patients who undergo ASA have more than twice the risk of permanent pacemaker implantation and 5 times higher risk of the need for additional septal reduction therapy, compared to those who undergo myectomy. Also, other systematic analysis [50] confirmed no significant difference in symptom relief between the two approaches. ASA was as safe as myectomy with regard to SCD and short-term and long-term mortality. The benefit from ASA has been very recently confirmed by Veselka et al. [51] studies, suggesting that, in patients with obstructive HCM and important symptoms who underwent ASA, long-term survival after the procedure did not differ significantly from that of the general population. Additionally, Veselka et al. [52] have documented that results of the first European multicenter study demonstrated that real-world early outcomes of ASA patients were better than had been reported in earlier observations from the first decade after ASA introduction. Finally, in the newest, large-scale multinational Euro-ASA registry with 1275 patients, Veselka et al. [53] have demonstrated low periprocedural and long-term mortality after ASA. This intervention provided significant relief of symptoms and a reduction of LVOT obstruction.

The American College of Cardiology Foundation/American Heart Association guidelines reserve ASA for elderly patients and patients with serious comorbidities but recent study [54] changes the clinical perspective. Accordingly, ASA is similarly effective for reduction of symptoms in young and elderly patients; however, younger patients have a lower risk of procedure-related atrioventricular conduction disturbances. The long-term mortality rate and risk of adverse arrhythmic events following ASA are low, in both young and elderly patients, and are comparable to age-matched nonobstructive HCM patients.

From pathophysiological point of view, LVOT obstruction and subsequent mitral regurgitation appear to further impair left atrial functional mechanics [55]. Importantly, septal myectomy has significantly reduced left atrial volumes, paralleled by an improvement in hemodynamic function [55].

## 7. From Technical Point of View in Both Surgical and ASA Procedures Novel Invasive Interventions Have Been Very Recently Developed 

Myectomy cannot be performed in 5–15%, due to technical difficulties. Also ASA methodology has several limitations, which are currently overcome. The newest 10 innovative procedures have been discussed.

### 7.1. 1st Innovation

Modification of surgical approach: exposure of the basal and midventricular septum through the aortic root to relieve obstruction in HCM can be challenging. Inadequate myectomy will lead to persistent symptoms and disability. Adequate exposure of the obstructive left ventricular septum is of paramount importance in primary and secondary corrected myectomy. In selected patients, either at primary myectomy or at remyectomy, septal excision can be approached through a left ventricular transapical incision when transaortic exposure is inadequate [56]. Transapical myectomy may be suitable approach for severe midventricular obstructive HCM [57].

### 7.2. 2nd Innovation

In surgical approach a very novel intervention is transaortic chordal cutting with mitral valve repair for obstructive HCM with mild septal hypertrophy [58]. This procedure relieves heart failure symptoms, abolishes LVOT gradient, and avoids mitral valve replacement in patients with obstructive HCM and mild septal thickness.

### 7.3. 3rd Innovation

There is strong evidence that treating the mitral valve abnormalities is a key feature of obstructive HCM. Investigators [59] successfully corrected both the anterior and posterior leaflet size and geometry. The proposed technique is double-stage procedure: first septal resection through the aortic valve and the detached anterior leaflet of the mitral valve and second mitral valve repair by reducing posterior leaflet height (leaflet resection or artificial neochordae) and increasing anterior leaflet height with pericardial patch.

### 7.4. 4th Innovation

The new concept has been also developed on percutaneous technique [60–62]. It was reported on catheter-based treatment of LVOT obstruction, targeting primarily a systolic anterior motion of the anterior mitral leaflet in obstructive HOCM. A patient was successfully treated with the MitraClip [60] two years after septal myectomy, in conjunction with mitral valve repair. The results prove the concept that systolic anterior motion (SAM) is clearly involved in gradient formation and is more than an epiphenomenon in obstructive HCM. Thus, SAM-induced subaortic obstruction might be a target for MitraClip implantation.

### 7.5. 5th Innovation

The transradial approach using a sheathless guiding catheter has appeared feasible and safe for ASA [63].

### 7.6. 6th Innovation

Peri-intervention monitoring of labile LVOT gradient is very practical. The intravenous nitroglycerin test during ASA is a useful method for rapidly confirming acute reduction of the latent gradient after the ASA procedure, and the outcome of ASA for labile obstruction was favorable [64].

### 7.7. 7th Innovation

**Figure 4:**
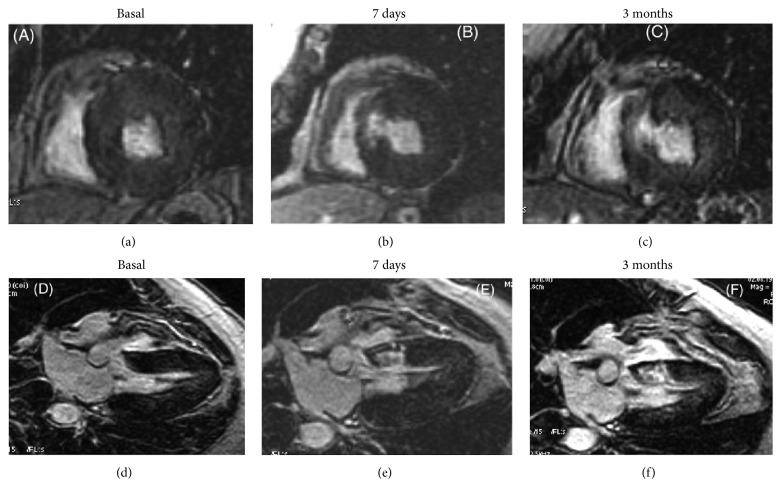
Cardiac magnetic resonance imaging before and after septal reduction therapy (myocardial scar after blockage of septal perforator artery by coil). Example of extension of delayed contrast-enhanced images of patchy areas of hyperenhanced myocardial in the interventricular septum 7 days (b, e) and 3 months (c, f) when compared with baseline (a, d). (a–c) Shot axis view. (d–f) Three-chamber view (reprinted with permission from [65]).

 It consists in nonsurgical septal myocardial reduction (ischemic scar by coil embolization into septal perforator artery) [65] (Figure 4).

### 7.8. 8th Innovation

Glue septal ablation is a promising technique for the reducing LVOT obstruction [66]. Glue seems to be superior to alcohol due to some intrinsic advantageous properties of glue such as immediate polymerization which prevents the leak into the left anterior descending coronary artery. Glue septal ablation technique is particularly useful in patients with collaterals to the right coronary artery in whom alcohol ablation is risky and contraindicated.

### 7.9. 9th Innovation

Improved renal function in a patient with obstructive HCM after multidetector computed tomography-guided ASA was witnessed [67].

### 7.10. 10th Innovation

 Cooper et al. [68] have proposed that method of delivering percutaneous tissue damage to the septum that is not reliant on coronary anatomy is desirable. To directly ablate the interventricular septum at the mitral valve systolic, anterior motion-septal contact point using radiofrequency energy guided by CARTOSound was shown. The authors have postulated that radiofrequency ablation using CARTOSound guidance is accurate and effective in treating LVOT gradients in HCM.

## 8. Phenocopies with LVOT Gradient with Successful ASA Treatment 

Useful for everyday practice are reports of invasive reduction of LVOT gradient in diseases mimicking HCM as phenocopies. This problem is associated with storage and infiltrative disease. Using ASA method, LVOT gradient was effectively reduced in Fabry disease [69] and amyloidosis [70]. Surgical correction was successfully performed in Noonan disease [71]. Septal myectomy may be a viable option to relieve symptoms and interrupt progression of heart disease also in selected Friedreich's ataxia patients [72].

## 9. Conclusion

From a practical point of view, the effective monitoring, including biochemical biomarkers indicating reduction of LVOT gradient, is important because this parameter has become a risk factor for sudden death in the 2014 ESC guideline [73]. In this context, the maximized physiological LVOT gradient provocation stimulus seems to be an important diagnostic element. Novel invasive therapeutic techniques have been recently dynamically developed, providing attractive treatment opportunities.
